# Can Linked Electronic Medical Record and Administrative Data Help Us Identify Those Living with Frailty?

**DOI:** 10.23889/ijpds.v5i1.1343

**Published:** 2020-08-13

**Authors:** ST Wong, A Katz, T Williamson, A Singer, S Peterson, C Taylor, M Price, R McCracken, M Thandi

**Affiliations:** 1 University of British Columbia, 2211 Wesbrook Mall, Vancouver, BC, V6T 2B5; 2 University of Manitoba, 408-727 McDermot Ave, Winnipeg, Mb, R3E 3P5; 3 University of Calgary, 3330 Hospital Drive NW, Calgary, Alberta, T2N 4N1

## Abstract

**Introduction:**

Frailty is a complex condition that affects many aspects of patients’ wellbeing and health outcomes.

**Objectives:**

We used available Electronic Medical Record (EMR) and administrative data to determine definitions of frailty. We also examined whether there were differences in demographics or health conditions among those identified as frail in either the EMR or administrative data.

**Methods:**

EMR and administrative data were linked in British Columbia (BC) and Manitoba (MB) to identify those aged 65 years and older who were frail. The EMR data were obtained from the Canadian Primary Care Sentinel Surveillance Network (CPCSSN) and the administrative data (e.g. billing, hospitalizations) was obtained from Population Data BC and the Manitoba Population Research Data Repository. Sociodemographic characteristics, risk factors, prescribed medications, use and costs of healthcare are described for those identified as frail.

**Results:**

Sociodemographic and utilization differences were found among those identified as frail from the EMR compared to those in the administrative data. Among those who were >65 years, who had a record in both EMR and administrative data, 5%-8% (n=191 of 3,553, BC; n=2,396 of 29,382, MB) were identified as frail. There was a higher likelihood of being frail with increasing age and being a woman. In BC and MB, those identified as frail in both data sources have approximately twice the number of contacts with primary care (n=20 vs. n=10) and more days in hospital (n=7.2 vs. n=1.9 in BC; n=9.8 vs. n=2.8 in MB) compared to those who are not frail; 27% (BC) and 14% (MB) of those identified as frail in 2014 died in 2015.

**Conclusions:**

Identifying frailty using EMR data is particularly challenging because many functional deficits are not routinely recorded in structured data fields. Our results suggest frailty can be captured along a continuum using both EMR and administrative data.

## Introduction

Over the past decade and a half, public spending on health care has doubled, accounting for nearly half of all provincial government expenditures [1]. High users of the healthcare system make up about 5% of the population but account for the majority (60%) of healthcare utilization and costs [2-7]. Approximately 40% of all health spending is for hospital care [1, 8]; in part, these hospital costs are due to those who are severely frail (completely dependent for personal care) to terminally ill (approaching the end of life) [9].

Frailty is considered a complex medical syndrome with numerous causes characterized by reduced strength, endurance and physiological function which results in reduced ability to recover following a stressful event, increased vulnerability to functional decline, dependence and/or adverse outcomes such as falls, disability, delirium, and death [10-12, 9, 13]. These adverse health events translate to increasing costs for the overall healthcare system. The majority of the 250,000 who die annually in Canada [14] are considered frail; and, the number of deaths is expected to double in the next 40 years due to the proportion of elderly rising to 25% of the population by 2030 [15].

The gold standard for assessing frailty is the comprehensive geriatric assessment (CGA) [10]. Periodic health assessments using a validated CGA are associated with better health outcomes for those aged 65 years and older [16]; however, this approach is time and resource intensive, and is not always practical in the primary care setting. The Canadian Study of Health and Aging Clinical Frailty Scale (CSHA-CFS), developed by Rockwood et al. (2005) [9], is one of the most widely accepted tool to identify frailty in Canada. It uses clinical judgment to assign an individual a score from 1 (very fit) to 9 (terminally ill), based on descriptions of comorbidity, cognitive impairment and disability (see Appendix A) [17, 9]. Scores of 5 and above characterize the clinical syndrome of frailty. This scale was found to be simple and easy to use and highly correlated (r = 0.80) with other established Frailty Index tools that rely on a CGA [17]. Each categorical increment of the Clinical Frailty Scale significantly increased the medium-term risks of death (21.2% within about 70 months, 95% confidence interval [CI] 12.5%–30.6%) and entry into an institution (23.9%, 95% CI 8.8%–41.2%), in multivariable models that adjusted for age, sex and education [17].

With an aging population and rising healthcare costs, it is recognized that more can be done at an individual and population level to delay frailty and compress morbidity, to improve functional ability until close to the time of death [18]. There is some urgency in developing more routine and standardized frailty screening and assessment [19], particularly in primary care. However, there remains a lack of consensus about how to best carry out this screening and assessment [20], leading to a proliferation of various scales and indices to measure frailty [21].

Preventing, reducing or delaying frailty has the potential to help mitigate the burden on individuals and society. Yet, assessing and reliably identifying frailty remains a controversial topic and relevant intervention research is still in its infancy. Early identification of frailty needs to begin in primary care [22]; however, tools and resources are required to assist clinicians in addressing the health and healthcare needs of those who are frail. Primary care clinicians (e.g. family physicians, nurse practitioners) are well placed to work with and meet the needs of individuals over 65 years who may not be managing or coping well with their health and/or social needs [23], putting them at an increased risk of frailty. Differences between these individuals and those who are managing well cannot be accounted for by age, comorbidities or medical treatments (e.g. polypharmacy) alone; thus, accurate detection of frailty in practice and at a population level is needed. Identification of frailty at a population level in primary care could contribute to population level planning. The main goals of caring for those who are frail in primary care are to improve function and quality of life while avoiding unnecessary admission to hospital or long-term care [24] or to delay increasing severity of frailty [25]. Moreover, identifying those who are at risk of becoming frail in primary care could enable targeted communications with patients and families. Targeted community-based resources in order to address the needs of both patients and their caregivers can then be implemented based on early identification of frailty.

The objective of this work was to: 1) use available data to explore and determine definitions of frailty that can be used in EMR and administrative data sources; and 2) examine whether there are differences in demographics or health conditions among those identified as frail in either the EMR or administrative data. 

## Methods

This cross-sectional descriptive study used linked EMR and administrative data in British Columbia (BC) and Manitoba (MB) to identify those aged 65 years and older who were frail. 

### Data Sources

#### EMR Data:

The pan-Canadian Primary Care Sentinel Surveillance Network (CPCSSN) consists of a network of networks across Canada [26]. There are over 1250 primary care clinicians (family physicians and nurse practitioners) and almost 2 million patients who provide their de-identified data to CPCSSN for the purposes of research, chronic disease surveillance and quality improvement across Canada [27]. The participating clinicians are more likely to be younger and female than a general sample of primary care clinicians, however, patients included in the CPCSSN are representative of those that visit primary care in Canada [28]. Patients of consenting sentinel providers can decline to participate in the CPCSSN (less than 0.01%) and their patient records are then excluded from this Pan-Canadian clinical data repository. All networks have received research ethics board approval from their institution for collecting this information.

The EMR data for this study were obtained from the British Columbia (BC) and Manitoba (MB) CPCSSN nodes. At the time of this work, BC and Manitoba nodes had 179 primary care clinicians, 46 in BC and 133 in MB. 

#### Administrative Data:

Administrative data were accessed from Population Data BC and the Manitoba Population Research Data Repository housed at the Centre for Health Policy (MCHP). The following files were accessed in both BC and Manitoba: CPCSSN data, Medical services billings (Medical Services Plan, BC; Manitoba medical claims), Consolidation file (BC)/Health insurance registry (MB) for identification of individual characteristics (e.g. age, sex, neighborhood), Census data for income quintiles, Hospital Discharge abstracts database, Vital Statistics, and the Pharmanet (BC)/Drug program information network (MB) data file. Manitoba also accessed the long-term care utilization data from Manitoba Health and home care minimum dataset assessment and utilization data through the MB support services payroll file.

#### Data linkage:

Once data access requests within each province were approved by the university ethics boards and appropriate entities who oversee the use of administrative data (e.g. BC Ministry of Health, MB Health Information Privacy Committee), EMR and administrative data were linked by Population Data BC and MCHP, respectively. Data linkage processes differ between the two jurisdictions, but both used a deterministic approach in this study.

In BC, Population Data BC has a repository of linkable administrative data files with scrambled unique identifiers. The BC CPCSSN completed a study specific extraction to create a linkage file containing CPCSSN ID and personal health number (PHN). This file was provided to Population Data BC in order to link the EMR and all administrative files. Once the data were successfully linked, using deterministic linkage and requiring exact match on PHN, the project-specific IDs were sent to BC CPCSSN whereupon the CPCSSN IDs were stripped from the CPCSSN data files and replaced with project-specific IDs. The CPCSSN data files were then sent to Population Data BC. Population Data BC prepared the administrative data with the same scrambled project-specific IDs. The de-identified data files were made available to the research team through a secure research environment within Population Data BC.

In MB, both the de-identified CPCSSN and administrative data files in the Repository had unique scrambled identifiers that included facilitated linkage at the person level. The MB CPCSSN completed a separate extraction to create a linkage file. This file was sent to the MB Information Management and Analytics Branch of the Ministry of Health, where they created a scrambled identifier. The scrambled identifiers were sent to MCHP to complete the deterministic linkage between the CPCSSN and administrative files. The dataset was then stripped of the scrambled identifier and the de-identified dataset was made available to the MCHP analyst who works with the research team.

The data linkage processes outlined by MCHP and Population Data BC allow for the secure and consistent linking of data across multiple data sets, and follow high quality guidelines to mitigate biases and ensure the highest level of matching possible. Detailed descriptions of data linkage procedures for MCHP and Population Data BC are available at https://ijpds.org/article/view/1131 and https://ijpds.org/article/view/1133, respectively. The linkage rate for this study was 97% in BC and 96% in MB. It is important to note that combining the administrative data across provinces is not allowable. Therefore, we use a distributed analyses process where we agree on the analyses and then it is carried out similarly in the two provinces. All procedures were approved by the University of British Columbia and University of Manitoba ethics review boards. 

#### Frailty Case Definition – EMR:

The research team developed and validated a case definition for frailty screening using the Southern Alberta CPCSSN node, described in detail elsewhere [29]. Briefly, 52 family physicians applied the Canadian Study of Health and Aging Clinical Frailty Scale [9] to randomly selected charts of their own patients in order to form a reference set (n=875; n=150 considered frail). The reference set was fed into a machine learning algorithm. We used Chi-square Automatic Interaction Detection (CHAID) supervised machine learning to create the algorithm; a decision tree was created from the algorithm by conducting multiple chi-square tests on potential features for defining frailty. A bootstrap validation technique was used to determine optimal complexity parameters that minimized the misclassification rate. Final validity estimates were calculated using 10-fold cross-validation. The CHAID machine learning took into consideration over 11,000 features (e.g. n=2,175 ICD-9 billing codes, n=4,870 cause for initial visit, n=2,438 ATC (Anatomical Therapeutic Chemical) codes from medication table).

The frailty EMR definition uses a combination of International Classification of Diseases 9th Edition (ICD-9) diagnosis (290 for dementia), ATC codes from the medication table (prescription for vitamins and/or furosemide) and free text (obstruction as a key word) from the billing data. The frailty EMR case definition has less than adequate sensitivity of 28% (95% CI (Confidence Interval): 21.0-36.0) but adequate specificity of 94% (95% CI: 93.0-96.0), a positive predictive value of 53% (95% CI: 42.0-64.0), and a negative predictive value of 86% (95% CI: 83.1-88.0).

The frailty EMR definition was applied to the EMR data in both Manitoba and BC, on 6 years of data (2009-2014). The EMR data captures only visits with primary care providers, and not visits with specialists, hospital contacts or long-term care records. Thus, a longer time period (than for the administrative data definition, below) was considered necessary for frailty criteria to be found if applicable.

#### Frailty case definition – Administrative:

A modified definition of frailty was developed based on previous work [30] and the BC Ministry of Health [31]. We applied three identification rules in patients ≥65: (1) Resident in a long-term care or assisted living facility; (2) Terminally ill; and (3) At least two indices from the modified Edmonton Frail Scale [32]. Patients aged 65+ meeting at least one of the three identification rules in the 2014 administrative data were considered to be frail. Not all datasets and variables were consistently available in each province, therefore the research teams in each province used their best available sources to identify the criteria for each identification rule (see supplemental material).

#### Inclusion criteria:

All patients who were aged 65+ on Jan 1, 2014 who had visited a primary care provider between Jan. 1, 2013 and Dec. 31, 2014 in the EMR data were included in this study. Patients who met these criteria but who were registered with the province for health care for <75% of the time they were alive in 2014 were excluded. This was done to ensure that patients who remained in the study were present in the province for a sufficient amount of time for the administrative frailty criteria to be found if applicable.

#### Variables of Interest:

We examined the patient characteristics of mutually exclusive groups, specifically those identified as frail in the EMR, administrative, and EMR + administrative frailty including; age (65-74 years, 75-84 years, 85+ years), sex (female, male), neighborhood income quintile, geographic location (rural, urban), blood pressure and body mass index (BMI). Neighborhood income quintiles derived from census data were used as a measure of SES ranked from 1 (lowest) to 5 (highest). In Manitoba, patients’ residence in rural or urban areas was determined using the first three digits of their postal code, also known as the forward sortation area. Following Canada Post’s procedure for classification, we coded residence as rural if there was a value of zero in the second digit of their forward sortation areas and urban for those with all other values. In British Columbia, patients’ residence was determined using statistical area classification types via Population Data BC [33]. Blood pressure (systolic and diastolic), derived from EMR data, was calculated based on the three most recent readings available. BMI was also derived from EMR data.

We also examined whether those identified as frail had any of the other chronic conditions for which the CPCSSN has a validated case definition (hypertension, diabetes, chronic obstructive pulmonary disease, osteoarthritis, dementia, depression, epilepsy, and parkinsonismAfter we had received our data, CPCSSN has since added three more validated case definitions (Chronic Kidney Disease, Herpes Zoster, and Pediatric Asthma). These were not included in our analysis.) as well as the number of chronic conditions. In order to calculate mean number of these same comorbidities in administrative data, we required one hospital diagnosis (ICD-10-CA) and/or at least two physician (ICD9) diagnoses within a rolling two-year period using data from 2009-2014 for the person to be classified with each condition. The exception was depression, for which at least two physician diagnoses had to be within a rolling one-year period (and/or one hospitalization), and, due to the sometimes transient or waxing/waning nature of the condition, if the initial diagnosis was prior to 2014, there needed to be evidence of treatment (any anti-depressant medication, physician visit or hospitalization related to depression) in 2014 to remain classified with depression.

#### Analysis:

Data were analyzed using three mutually exclusive groups: frail in EMR, frail in administrative, and frail in EMR + administrative data. We examined the prevalence of frailty among those who had visited a primary care provider in the EMR data in the last two years. For descriptive analyses we dichotomized income quintiles into high (3, 4 and 5) and low (1 and 2) SES. Descriptive statistics were used to calculate the mean number of comorbidities. We used appropriate statistics to examine whether there were differences in characteristics among those who were identified as frail between the EMR and administrative data. Demographic and other characteristics were examined using 2014 data and use/cost analysis were completed with 2015 data in order to examine the effect of frailty on the next year of healthcare service use. All analyses were done using SAS 9.4.

## Results

The EMR data for a total of 33,663 patients aged 65 years and older was linked to administrative data in British Columbia and Manitoba. We identified about 1% of patients as frail in both the EMR and administrative data in both British Columbia and Manitoba (Table 1).

**Table 1: Patients aged 65 years and older identified in EMR and administrative data (2014) as frail:** 

Cases of Frail Patients	British Columbia linked data	Manitoba linked data
	N=4978	N=28,685
Identified in EMR data only, n (%)	91 (1.8)	1589 (5.5)
Identified in Administrative data only, n (%)	517 (10.4)	1821 (6.3)
Identified in Both (EMR & admin data), n (%)	70 (1.4)	307 (1.1)

Table 2 below shows there was a higher likelihood of being frail with increasing age, being female and if the patient lived in an urban area. Across BC and MB, we found different characteristics of those who were identified as frail. There were more frail patients who were classified in the higher income quintiles, particularly in BC. The blood pressures of those identified as frail in Manitoba were in the hypotensive systolic range although this pattern was not observed among the patients in either BC cohort. The diastolic blood pressures of all patients identified as being frail were fairly consistent with an average range of 70.9-71.7 mmHg. Since blood pressure readings were obtained from EMR data linked with the administrative data, this explains the absence of almost 50% of the blood pressure readings in the “frail in administrative data only” compared to those with almost 100% of BP values being present in the “frail in EMR data only”. If patients were identified as frail using either the EMR algorithm alone or with both the EMR and administrative data algorithms, then we saw a pattern of increasing numbers of chronic conditions.

**Table 2: Patient characteristics of those identified as frail:** **Note:** Chi-squared tests for categorical variables and t-test for continuous variables, comparing the Frail cases in EMR data to the Frail cases in the admin data only, were not significantly different with the following exceptions: In BC: chronic conditions (admin data) +p<0.05; geographic location +p<0.01; chronic conditions (EMR data) +p<0.0001. In MB: income quintile *p<0.05; age group *p<0.0001; geographic location (urban/rural) *p<0.0001; chronic conditions (admin data) *p<0.0001; chronic conditions (EMR data) *p<0.0001 Note:** Blood pressure results are missing for less than 9% of Cases in EMR data, but there is a higher percent who had no blood pressure readings in 2013-2014 for the other categories. The percent with no blood pressure readings are: Cases in Admin data: BC 56.5%; MB 58.5%, Cases in Both: BC 27.1%; MB 21.5%. +counts chronic conditions from the CPCSSN 8 validated case definitions (Chronic Obstructive Pulmonary Disease, Dementia, Depression, Diabetes Mellitus, Epilepsy, Hypertension, Osteoarthritis, Parkinson’s Disease)

		Cases in British Columbia		Cases in Manitoba		Cases in Both EMR and Admin Data	
N(%)		EMR: British Columbia N=91	Admin: British Columbia N=517	EMR: Manitoba N=1589	Admin: Manitoba N=1821	British Columbia N=70	Manitoba N=307
Age(*)							
	65-74	27 (29.7)	122 (23.6)	519 (32.7)	270 (14.8)	5 (7.1)	42 (13.7)
	75-84	35 (38.5)	185 (35.8)	610 (38.4)	610 (33.5)	20 (28.6)	94 (30.6)
	85+	29 (31.9)	210 (40.6)	460 (28.9)	941 (51.6)	45 (64.3)	171 (55.7)
Sex							
	Female	55 (60.4)	300 (58.0)	1,006 (63.3)	1,163 (63.9)	51 (72.9)	196 (63.8)
	Male	36 (39.6)	217 (42.0)	583 (36.7)	658 (36.1)	19 (27.1)	111 (36.2)
Income (*)							
	High (3rd – 5th quintile)	58 (63.7)	281 (54.4)	810 (51.4)	714 (47.8)	41 (58.6)	142 (50.5)
	Low (1st – 2nd quintile)	33 (36.3)	236 (45.6)	766 (48.6)	780 (52.2)	29 (41.4)	139 (49.5)
Geographic Location (+*)							
	Rural	35 (38.5)	118 (22.8)	692 (43.5)	548 (30.1)	26 (37.1)	105 (34.2)
	Urban	56 (61.5)	399 (77.2)	897 (56.5)	1,273 (69.9)	44 (62.9)	202 (65.8)
*Blood Pressure							
	Systolic	130.8 (18.6)	128.9 (16.3)	98.3 (44.0)	100.5 (40.3)	131.6 (15.6)	102.7 (40.5)
+Chronic Condition from admin data (+*)							
	0-1	20 (22.0)	128 (24.8)	269 (16.9)	428 (23.5)	16 (22.9)	32 (10.4)
	2	46 (50.5)	185 (35.8)	461 (29.0)	586 (32.2)	24 (34.3)	55 (17.9)
	3+	25 (27.5)	204 (39.5)	859 (54.1)	807 (44.3)	30 (42.9)	216 (70.4)
+Chronic conditions from EMR data (+*)							
	0-1	18 (19.8)	285 (55.1)	256 (16.1)	843 (46.3)	10 (14.3)	32 (10.4)
	2	31 (34.1)	109 (21.1)	412 (25.9)	442 (24.3)	25 (35.7)	62 (20.2)
	3+	42 (46.2)	123 (23.8)	921 (58.0)	536 (29.4)	35 (50.0)	213 (69.4)

**Table 3: 2015 costs of healthcare and deaths of patients (65+) identified as frail:** **Note:** The decrease in n from table 2 is due mostly to deaths in 2014 (plus very small number lost-to-follow-up (i.e. moving out of province)).* Chi-squared tests for categorical variables and t-test for continuous variables, comparing the Frail cases in EMR data only to the Frail cases in the admin data only, were not significantly different with the following exceptions. In BC: total cost of other spending (laboratory and imaging) +p<0.05, died in 2015 +p<0.01. In MB: total cost of GP care *p<0.05; cost of acute hospital care *p<0.05; number of days in a hospital *p<0.01; total cost of specialist care *p<0.0001; total cost of other spending (lab and imaging) *p<0.0001; total cost of physician care *p<0.0001; number of acute hospitalizations *p<0.0001; polypharmacy *p<0.0001; died in 2015 *p<0.0001. All costs are in $CAD

	Cases in British Columbia		Cases in Manitoba		Cases in Both EMR and Admin Data	
Mean (SD)	EMR: British Columbia N=91	Admin: British Columbia N=387	EMR: Manitoba N=1553	Admin: Manitoba N=1319	British Columbia N=56	Manitoba N=216
Number of contacts with GP	21.1 (18.6)	20.8 (18.5)	17.1 (14.6)	17.7 (14.9)	21.3 (22.1)	20.5 (15.6)
Total annual cost of GP care (*)	1117.6 (1073.1)	998.0 (1138.4)	831.4 (1087.5)	743.3 (1002.1)	928.8 (1226.4)	989.1 (1176.7)
Total cost of specialist care (medical & surgical) (*)	1092.5 (1971.1)	910.9 (1619.6)	922.8 (1843.9)	402.0 (1042.1)	477.8 (902.8)	610.7 (1694.8)
Total cost of other spending (laboratory and imaging) (+*)	434.0 (493.0)	299.5 (403.6)	271.9 (297.6)	137.9 (223.3)	233.3 (285.6)	199.5 (282.2)
Total cost of physician care (*)	2644.2 (2719.2)	2208.4 (2564.3)	2169.4 (2733.4)	1359.3 (1935.3)	1639.9 (1907.1)	1884.2 (2584.4)
Cost of acute hospital care (*)	5607.6(14089.8)	7805.4 (19873.5)	8169.8 (23881.2)	6052.1 (25064.4)	7212.8 (16191.6)	11877.6 (33336.5)
Number of acute hospitalizations in a year, per 100 people (*)	59.3 (139.8)	58.1 (106.6)	47.4 (88.9)	27.9 (67.2)	58.9 (100.5)	48.2 (84.6)
Total cost of annual prescriptions	2618.9 (2459.0)	2276.9 (3382.9)	2371.8 (2986.0)	2279.9 (2431.7)	1744.4 (2207.9)	2584.2 (4128.2)
Polypharmacy (> 5 prescribed medications), n (%) (*)	80 (87.9)	311 (80.4)	1221 (78.6)	649 (49.2)	42 (75.0)	144 (66.7)
Number of days in a hospital in a year (*)	6.1 (15.4)	8.4 (23.6)	8.5 (26.8)	5.6 (23.3)	7.2 (17.5)	9.8 (24.8)
Died in 2015, n (%) (+*)	6 (6.6)	77 (19.9)	36 (2.3)	136 (10.3)	15 (26.8)	30 (13.9)

In both BC and MB, those identified as frail in both data sources have approximately twice the number of contacts (n=20 vs. n=10) with primary care and more days in hospital compared to those who are not frail (n=7.2 vs. n=1.9 in BC; n=9.8 vs. n=2.8 in MB). In both BC and MB, cases where people were identified as frail in the EMR data had higher use and costs for primary and specialist care and other kinds of tests such as laboratory and imaging compared to those identified as frail in the administrative data. The mean cost of hospital care was higher in BC but lower in MB among those identified as frail in the administrative data compared to those identified as frail in the EMR data. Among those who were identified as frail (2014) in both the EMR and administrative data, 15 (27%) in BC and 30 (14%) in MB died in 2015.

## Discussion

This study is the first in Canada to use linked EMR and administrative data to examine frailty. We used these data to determine whether frailty could be identified in similar ways using both data sources. The identification of frailty using these routinely collected data remains elusive. Many chronic conditions have been defined with clear biomedical markers such as HbA1c for diabetes, creatinine clearance for renal failure, echocardiology for congestive heart failure, or blood pressure for hypertension. However, frailty is a clinical syndrome comprised of a variety of functional deficits ranging from reduced strength and endurance to a longer time to recover following a stressful event. Many of these deficits are not routinely recorded in primary care notes nor would they show up in administrative data until a patient is severely frail (i.e. admitted to a nursing home). This poses a particular challenge for identifying mild or moderately frail individuals or those at risk of experiencing increasing frailty, especially amongst older adults whose decline in functional status is also considered a natural part of aging [34-35].

This study is unique in attempting to use routinely collected EMR data to identify frailty. Our EMR definition is under-capturing the true prevalence of frailty in patients cared for in primary care settings as evidenced by the low sensitivity of the definition. Increasing the sensitivity of the primary care EMR frailty definition will likely need supplemental assessment data such as the Clinical Frailty Scale [5], 3-metre gait speed test [36], or other tools since exclusively using routinely collected primary care EMR data are not sufficient. It may also be possible that advances in natural language processing may support enhanced identification of frailty, leveraging the application of artificial intelligence methodologies to narrative notes, although this technology has not yet been developed or validated. Recent work by Rosenberg, et al. (2019) [22] suggests that the CFS could be administered as a quick screening method if further frailty assessment is needed. They showed that patients with CFS levels 5-7 had lower functional health status, lower grip strength, nutrition and cognition and increased depression compared to those with CFS levels 2-4.

The differences between those identified as frail in only the EMR or only the administrative data is likely occurring for two reasons. First, those who were identified as frail in only the administrative data were terminally ill or lived in a long-term care facility/assisted living and had some indication of major functional decline [30]. It is likely that these people are living in nursing homes and therefore data relating to their care is not being captured in community based primary care EMR data. Second, frailty is likely a continuum as depicted by the CFS where individuals decline in their functional abilities over time. Therefore, we would expect the administrative algorithm to miss those who are earlier along the continuum of frailty. Linking EMR and administrative data could more accurately identify the range of people who are frail and potentially provide a broader understanding of the impacts of increasing frailty.

The costs of health care are higher for those identified as frail only in the EMR compared to those only in the administrative data in both jurisdictions, although statistically significant only in Manitoba. It could be that those identified as frail in primary care are earlier along a continuum for frailty [22]. These patients have more family and specialist physician visits and more laboratory and diagnostic tests, which suggests they are still gathering prognostic information and must decide the intensity of medical intervention or community and institutional supports. The lower mortality rate amongst those identified as frail in only the EMR data could also indicate that the different frailty definitions are identifying people along a continuum of frailty. It is also likely that those identified as frail only in the administrative data are living in nursing homes and their care is not captured in community based primary care EMRs.

Limitations. This study has several limitations that warrant consideration. The primary care EMR contains information collected by primary care providers for the primary purpose of clinical care (e.g. medications prescribed, billing and encounter data) whereas the administrative data also contains these data for specialists and hospital data. We did not use ACG (adjusted clinical group) since frailty within the administrative data is very dependent on a dementia diagnosis. Indeed, we found that people with an ACG frailty flag was 67% and we had separately identified 62% with dementia suggesting a high amount of overlap. The number of chronic conditions reported here is dependent on the ones that could be reliably detected by the eight validated CPCSSN case definitions; we limited the chronic conditions that we counted in the administrative data to be the same as those identified in the EMR data. Therefore, the differences between the number of chronic conditions seen in administrative data compared to in the EMR data suggests there are differences in how these conditions are captured in the data sources and picked up by the disease-case algorithms. Home care and residential care data in BC were not available at the time of this study which means we are likely under-reporting costs due to missing the extremely frail requiring these services.

## Conclusion/Implications

Identifying frail community-based residents provides an important opportunity for the prevention of further deterioration, and to limit the risk of further complications, such as falls and poor diet. This study has demonstrated some of the potential uses and challenges in identifying this clinical syndrome from routinely collected data, whether it be extracted from EMRs, or based on administrative claims data. The addition of specific fields to the EMR of patients aged 65 or older may be an acceptable mechanism to ensure the availability of the required information to support improved clinical care of frail patients through earlier identification.

## Acknowledgments

We would like to acknowledge the family physicians in British Columbia and Manitoba that took part in completing the Rockwood Clinical Frailty Scale.

## Conflicts of Interest.

The authors declare that they have no conflict of interest.

## Ethics statement

Ethical approval was obtained from the University of British Columbia (REB# H18-01341) and the University of Manitoba (REB# HS19744 (H2016:187)) ethics review boards.

## List of Abbreviations

EMR	Electronic Medical Record
BC	British Columbia
MB	Manitoba
CPCSSN	Canadian Primary Care Sentinel Surveillance Network
CGA	Comprehensive Geriatric Assessment
CSHA-CFS	The Canadian Study of Health and Aging Clinical Frailty Scale
MCHP	Manitoba Population Research Data Repository housed at the Centre for Health Policy
CHAID	Chi-square Automatic Interaction Detection (CHAID)
ATC	Anatomical Therapeutic Chemical
ICD	International Classification of Diseases
GP	General Practitioner
ACG	Adjusted Clinical Group
